# Bio-Piezoelectric Ceramic Composites for Electroactive Implants—Biological Performance

**DOI:** 10.3390/biomimetics8040338

**Published:** 2023-08-01

**Authors:** Beatriz Ferreira Fernandes, Neusa Silva, Joana Faria Marques, Mariana Brito Da Cruz, Laura Tiainen, Michael Gasik, Óscar Carvalho, Filipe Samuel Silva, João Caramês, António Mata

**Affiliations:** 1 Oral Biology and Biochemistry Research Group—Unidade de Investigação em Ciências Orais e Biomédicas (UICOB), Faculdade de Medicina Dentária, Universidade de Lisboa, 1600-277 Lisboa, Portugal; 2 Department of Mechanical Engineering, Center for Microelectromechanical Systems (CMEMS), University of Minho, 4800-058 Guimarães, Portugal; 3 Department of Chemical and Metallurgical Engineering, Aalto University, 02780 Espoo, Finland; 4 Implant & Tissue Regeneration Group—Unidade de Investigação em Ciências Orais e Biomédicas (UICOB), LIBPhys-FTC UID/FIS/04559/2013, Faculdade de Medicina Dentária, Universidade de Lisboa, 1600-277 Lisboa, Portugal; 5 Oral Biology and Biochemistry Research Group—Unidade de Investigação em Ciências Orais e Biomédicas (UICOB), LIBPhys-FCT UID/FIS/04559/2013, Faculdade de Medicina Dentária, Universidade de Lisboa, 1600-277 Lisboa, Portugal; 6 CEMDBE—Cochrane Portugal, Faculdade de Medicina Dentária, Universidade de Lisboa, 1600-277 Lisboa, Portugal

**Keywords:** dental implants, zirconia, piezoelectric properties, barium titanate, osteoblasts, fibroblasts

## Abstract

Barium titanate (BaTiO_3_) piezoelectric ceramic may be a potential alternative for promoting osseointegration due to its piezoelectric properties similar to bone electric potentials generated in loading function. In this sense, the aim of this in vitro study was to evaluate the cellular response of human osteoblasts and gingival fibroblasts as well as the impact on *S. oralis* when in contact with BaTiO_3_ functionalized zirconia implant surfaces with piezoelectric properties. Zirconia discs with BaTiO_3_ were produced and contact poling (piezo activation) was performed. Osteoblasts (hFOB 1.19), fibroblasts (HGF hTERT) and *S. oralis* were culture on discs. Cell viability and morphology, cell differentiation markers, bacterial adhesion and growth were evaluated. The present study suggests that zirconia composite surfaces with the addition of piezoelectric BaTiO_3_ are not cytotoxic to peri-implant cells. Also, they seem to promote a faster initial osteoblast differentiation. Moreover, these surfaces may inhibit the growth of *S. oralis* by acting as a bacteriostatic agent over time. Although the piezoelectric properties do not affect the cellular inflammatory profile, they appear to enable the initial adhesion of bacteria, however this is not significant over the entire testing period. Furthermore, the addition of non-poled BaTiO_3_ to zirconia may have a potential reduction effect on IL-6 mediated-inflammatory activity in fibroblasts.

## 1. Introduction

Dental implants are a preferred rehabilitation option to replace missing teeth, restoring aesthetics and function [1]. The success of implants mostly depends on the osseointegration process [1,2,3,4], however this can be inhibited by the formation of a fibrous membrane between the implant surface and peri-implant tissue [5]. Despite the great progress in the field of implantology over recent decades, the search for materials that reduce osseointegration time and enhance peri-implant healing and thus enhance the predictability of this treatment modality remains a priority. Implant material and surface treatment are important factors for bone and mucosal connective tissue integration, as well as to reduce the healing period [4,6]. Titanium alloys (especially Ti-6Al-4V) are currently the most widely used material for dental implants due to their excellent mechanical and biological properties [7,8,9]. However, this material presents as disadvantages the aesthetics and the occurrence of allergic reactions and corrosion that leads to the release of ions and debris [7,8,9,10]. In view of these disadvantages, alternatives to titanium have been explored, namely ceramic materials such as zirconia [2].

Also, the surface treatment to improve the interface between dental implant and bone tissue has been extensively characterized in the literature [10]. A novel approach based on biochemical and physicochemical properties, namely mechanical and electrical stimulation, has been suggested to enhance the implant–tissue interface [4,11]. Wolff’s law describes the concept of piezoelectricity as being the ability of bone to adapt to mechanical stress, through the conversion of mechanical stimuli into biochemical signals at the cellular level that result in intracellular changes—mechanotransduction [11,12,13,14,15]. There are several studies in the literature with experimental and computational techniques that support the theory proposed by Wolff [16,17,18]. In vivo studies also suggest that the application of electrical stimulation to the implant site accelerates the initial phase of osseointegration, improves implant–tissue interface strength, and increases bone formation [19,20].

In this sense, piezoelectric ceramics could be an alternative for dental implant biofunctionalization, as they produce mechanically generated electric surface potentials, in other words mimicking the bone’s ability to generate electrical energy when subject to mechanical load [11,21]. Although piezoelectric ceramics are not currently used as implant material, in vitro studies indicate improved biocompatibility and bone-inductive ability on piezoelectric ceramic surfaces [11].

Barium titanate (BaTiO_3_) is a ferroelectric ceramic that belongs to the family of piezoelectric materials, and due to its widely described ability to produce micro-electric currents, it has been investigated for its osteoconduction and osteoinduction properties, accelerating the osteogenesis and providing better osseointegration [5,21,22]. Thus, BaTiO_3_ piezoelectric ceramic has been used to promote bone regeneration [21], and could be an alternative implant material for promoting osteogenic proliferation and differentiation and, consequently, accelerating osseointegration [5,21,22]. However, bacterial infections and biofilm formation represent significant challenges to the success of implant procedures, leading to investigations into the antibacterial properties of BaTiO_3_ piezoelectric ceramic as a potential alternative to antibiotics, which can be ineffective due to bacterial resistance. The recent literature has focused on understanding the microbial inhibition properties of the BaTiO_3_ piezoelectric ceramic in order to develop new strategies for preventing bacterial infections and enhancing the long-term success of implant procedures [23,24,25,26,27,28].

There are several in vitro studies in the literature with different cell lines of osteoblasts and using different techniques for cell behavior evaluation. However, to our knowledge, there are no studies with fibroblasts or *Streptococcus oralis* and using BaTiO_3_-functionalized zirconia as dental implant material [4,5,11,29]. In this in vitro study we evaluate the human fetal osteoblasts, human gingival fibroblasts, and *Streptococcus oralis* responses—considering cell viability, differentiation, inflammatory profile (peri-implant cells), colony-forming units and biomass (bacteria)—in contact with barium titanate-functionalized zirconia implant surfaces with piezoelectric properties. The general working hypothesis was that piezoelectric composites (contact polarization-activated BaTiO_3_-functionalized zirconia) would induce improve cell responses from osteoblasts and fibroblasts (higher viability and lower inflammatory marker secretion) and a lower bacterial adhesion when compared to unpoled (non-piezoelectric BaTiO_3_ zirconia) and pure zirconia.

## 2. Materials and Methods

### 2.1. Samples Processing

A total of 15 discs were manufactured for each group (Table 1) using the press-and-sintering technique from a commercial 3 mol% yttria-stabilized zirconia (Y_2_O_3_) (Tosoh Corporation^©^, Tokyo, Japan). A total of 5% of BaTiO_3_ powder (219-6A, Ferro^®^, Mayfield Heights, OH, USA) was added to test samples. The powders were mixed dry in WAB Turbula^®^ T2 C—3D shaker mixer (Willy A. Bachofen AG Maschinenfabrik, Muttenz, Switzerland).

Green bodies were pressed from 1.2 ± 0.01 g of powder mixture and pressed in an 18 mm diameter die, lightly powdered with zinc stearate (C36H70O4Zn) (Sigma-Aldrich^®^, St. Louis, MO, USA). An initial pressure of 0.5 MPa was held for around 2 s and released. Then, the final pressure of 200 MPa was applied for 4 min. The die was disassembled and cleaned with a wipe between each disk. The flat surfaces of the die were polished with MicroCut^®^ P4000 (Buehler, Ltd., Lake Bluff, IL, USA) silicon carbide grinding paper.

Samples were then sintered in air at 1380 °C for 2 h with heating and cooling rate of 5 °C/min (Zirkonzahn sintering furnace, Zirkonzahn^®^, Norcross, GA, USA). The sintered discs were ground with a resin-bonded diamond grinding disc (MD-Piano 120, Struers, Cleveland, OH, USA) to final dimensions of ±0.02 mm. For grain size measurements and cell tests the samples were polished to P4000 SiC grinding paper. Prior SEM, polished samples were thermally etched 30 min at 1200 °C, heating rate 8 °C/min and <12 °C/min cooling rate. Other samples were heat-treated after polishing as potential polishing induced monoclinic phase needed to be retransformed to tetragonal by 2 h at 600 °C in air.

In poled samples (P) contact poling or polarization (alignment of the individual dipole moments so that they all point in the same direction) was carried out in a silicon oil bath under DC 2 kV/mm electric field at 130 °C for 30 min followed with field cooling [30]. At the end, prior to mechanical as biological assays, all samples were ultrasonically cleaned in 100% ethanol.

### 2.2. Samples Characterization

#### 2.2.1. Microstructure

Samples were inspected with an optical microscope. For grain size measurements, Scanning Electron Microscopy (SEM) images were carried out in backscattered electron mode (SEM/BSE). The analysis was performed with FIJI freeware software MorphoLibJ IJPB-plugin and images were acquired at 1000×, 10,000× and 50,000× magnifications.

#### 2.2.2. Surface Roughness

Surface roughness was evaluated using Ra parameter—the arithmetic mean deviation of the assessed profile. The Ra roughness was measured using a mechanical 2D profilometer (Surftest SJ 201, Mitutoyo, Kanagawa, Japan) in accordance with ISO 4288:1996 standard. The measures were recorded on different regions always changing the scanning directions. The scanning speed used was 0.25 mm/s and 3 × 1.5 mm lines were scanned.

### 2.3. Cell Cultures

Human fetal osteoblasts (hFOB 1.19—CRL-11372^TM^; ATCC^®^, American Culture Collection, Manassas, VA, USA) were cultured in 75 cm^2^ culture flask (VWR^TM^, Radnor, PA, USA) in an atmosphere of 98% humidity and 5% CO_2_ at 37 °C with a culture medium constituted of a mixture (1:1 *v*/*v*) of Ham’s F12 Medium (Sigma-Aldrich^®^, St. Louis, MO, USA) and Dulbecco’s Modified Eagle’s Medium—DMEM (Biowhittaker^TM^, Lonza^TM^, Basel, Switzerland) supplemented with 10% bovine fetal serum (Biowest, Nuaillé, France) and 0.3 mg/mL G418 (InvivoGen, Toulouse, France).

Immortalized human gingival fibroblasts (hTERT—T0026; Applied Biological Materials Inc., Richmond, BC, Canada) were cultured under the same atmospheric conditions previously described for osteoblasts. The culture medium was constituted of Dulbecco’s Modified Eagle’s Medium (DMEM) (Biowhittaker^TM^, Lonza^TM^, Switzerland) with 10% of bovine fetal serum (Biowest, Nuaillé, France) and 1% of penicillin-streptomycin (Lonza^TM^, Basel, Switzerland).

Cells were trypsinized using trypsin-EDTA (Lonza^TM^, Basel, Switzerland) and cells were seeded on discs, distributed in 24-well culture plates (Corning, Corning, NY, USA) at a density of 1 × 10^4^ cells/well. All biological assays were conducted using a fifth passage. For each assay a negative control (cells cultured directly on treated polystyrene surface of the well) was used.

#### 2.3.1. Cell Viability Assay

Cell viability was measured using a commercial resazurin-based method (Cell Titer Blue^®^, Promega, Madison, WI, USA) according to the producer protocol. The conversion rate was measured as fluorescence intensity in arbitrary fluorescence units (AU) after 1, 3, 7 and 14 days of cell culture in contact with the discs. Fluorescence intensity was detected at an excitation wavelength of 530/30 nm and emission wavelength of 595/10 nm using a multimode microplate reader (VICTOR Nivo^TM^ HH3500, PerkinElmer^®^, Beaconsfield, UK). Each group had a final size of *n* = 15.

#### 2.3.2. Backscattered Electrons in Scanning Electron Microscopy

After 24 h of osteoblasts and fibroblasts culturing, samples were washed with phosphate-buffered saline (PBS) (VWR^®^, Radnor, PA, USA), and cells were fixed with 2.5% glutaraldehyde (VWR^®^, Radnor, PA, USA) for 1 h. A dehydration process was carried out using a serial dilution of ethanol. Samples were covered with an ultra-thin film (15 nm) of Au-Pd (80-20 weight%); using a high-resolution sputter coater coupled to an MTM-20 Cressington High-Resolution Thickness Controller (208 HR Cressington Company, Hertfordshire, UK). Samples were observed under ultra-high resolution Backscattered Electrons in Scanning Electron Microscopy (BSE/SEM)—FEI NOVA 200 Nano SEM (FEI, Hillsboro, OR, USA). Secondary images were performed at different magnifications (100, 120, 200, 500×), at an acceleration voltage of 10 kV. Image analysis was performed by two calibrated researchers, focusing on cell morphology, spreading and the establishment of early contact with materials.

#### 2.3.3. Fluorescence Microscopy

At 24 h of culturing, samples cultured with osteoblasts and fibroblasts were washed with PBS (VWR^®^, Radnor, PA, USA). Cell fixation was performed using 4% formaldehyde solution for 10 min and stained with Phalloidin-iFluor 488 Reagent (ab176753, Abcam^®^, The Netherlands) and Propidium iodide (P4179, Sigma-Aldrich^®^, St. Louis, MO, USA). Fluorescence images were carried out at 493/517 nm and 535/617 nm wavelength using a Leica TCS SP5 confocal microscope (Leica Microsystems, Deerfield, IL, USA) coupled to LAS-AF LITE v2.0 software (Leica Microsystems, Deerfield, IL, USA).

#### 2.3.4. Alkaline Phosphatase (ALP) Activity

At 7 and 14 days of osteoblasts culturing, ALP activity was evaluated (*n* = 3) using a colorimetric enzymatic assay (ab83371, Abcam^®^, Cambridge, UK), following manufacturer instructions. Measurements were performed by colorimetric intensity at excitation wavelengths of 405/10 nm using a multimode microplates reader (VICTOR Nivo^TM^ HH3500, PerkinElmer^®^, Beaconsfield, UK). A standard curve was performed to calculate enzymatic activity and values were converted to μmol/min/mL.

#### 2.3.5. Interleukin 1β

Quantification of interleukin 1β of osteoblast and fibroblast cultures (*n* = 4) at 1 and 3 days were measured using Human IL-1 beta/IL-1F2 DuoSet ELISA Kit (R&D Systems, Inc., Minneapolis, MN, USA). The optical density was detected by multimode microplate reader (VICTOR Nivo^TM^ HH3500, PerkinElmer^®^, Beaconsfield, UK). The results were measured in units of absorbance (AU) relative to the light intensity values and were converted in pg/mL according to the standard curve.

#### 2.3.6. Interleukin 6

Interleukin 6 levels at 1 and 3 days of osteoblast and fibroblast cultures (*n* = 4) were performed by Human IL-6 DuoSet ELISA (R&D Systems, Inc., Minneapolis, MN, USA). The results were measured in absorbance units (AU) relative to the values of light intensity using a multimode microplate reader (VICTOR Nivo^TM^ HH3500, PerkinElmer^®^, Beaconsfield, UK) and were converted according to the standard curve carried out in pg/mL.

#### 2.3.7. Osteopontin

Osteopontin quantification at 3 and 7 days of osteoblast culture (*n* = 4) was performed by Human Osteopontin (OPN) DuoSet ELISA (R&D Systems, Inc., Minneapolis, MN, USA), by a technique of luminescence. The results were obtained in absorbance units (AU) using a multimode microplate reader (VICTOR Nivo^TM^ HH3500, PerkinElmer^®^, Beaconsfield, UK) and converted in pg/mL according to the standard curve.

#### 2.3.8. Mineralization Assay

After 7 days of incubation, discs with osteoblasts were washed 3 times with PBS (VWR^®^, Radnor, PA, USA) to remove non-adherent cells. Cells were fixed using 2.5% glutaraldehyde (VWR^®^, Radnor, PA, USA) for 1 h and then the discs were washed with PBS. Cell culture mineralization was evaluated using the OsteoImage^TM^ Mineralization Assay (PA-1503, Lonza, Morristown, NJ, USA). This assay consists of the fluorescent staining of extracellular mineral content deposited by cells, specifically hydroxyapatite. Mineralization-stained images were obtained at 492/529 nm excitation/emission wavelengths using a Leica TCS SP5 confocal microscope (Leica Microsystems, Deerfield, IL, USA) coupled to LAS-AF LITE v2.0 software (Leica Microsystems, Deerfield, IL, USA).

### 2.4. Bacterial Strain and Growth Conditions

To carry out this investigation, the *Streptococcus oralis* CECT 907T strain was cultured in anaerobic atmosphere on an enriched blood agar plate at 37 °C for 72 h (10% CO_2_, 10% H_2_ and balance N_2_). Subsequently, a single colony was grown in 15 mL of Brain–Heart Infusion Modified Medium (BHI-2) at 37 °C under anaerobic conditions. Upon achieving the exponential phase, the optical density (OD) at 550 nm of the suspension was measured using a Camspec M50 spectrophotometer to confirm the growth, and the OD was standardized to 0.4 for all subsequent experiments.

#### 2.4.1. Colony Forming Unit (CFU)

Sample discs from one of three groups, P, NP and YSZ, were randomly assigned to this experiment (*n* = 3). *Streptococcus oralis* CECT 907T strain were added to each disc in exponential phase, in a 24-well plate and incubated at 37 °C in anaerobic conditions. The effectiveness of the BaTiO_3_ piezoelectric ceramic as a bacteriostatic/bactericidal agent was determined by counting the Colony Forming Units (CFUs) on the discs after 1 and 2 days of culture. To determine the number of viable bacterial cells attached to the discs after biofilm formation, the discs were washed once with filtered phosphate-buffered saline (PBS) and placed in a Falcon tube with 3 mL of PBS. The tube was then vortexed for 1 min (16 rpm × 100), ultrasonicated for 4 min, and vortexed again for 2 min (16 rpm × 100). Ten-fold serial dilutions were performed until 10^−8^, and 20 μL of each dilution were plated in duplicate on supplemented blood agar plates, which were then incubated at 37 °C under anaerobic conditions. CFUs were counted after 24 h incubation. The experiment was repeated three times to ensure the validity and reliability of the results. Each trial was conducted using the same methods and procedures as the initial experiment.

#### 2.4.2. Biomass

After 1 and 2 days of culture, the bacterial biomass on the discs was assessed (*n* = 3). The samples were washed with sterile dH_2_O and allowed to dry inside a laminar flow chamber cabinet for 2 h. Then, 500 μL of 1% crystal violet was added and incubated for 20 min at room temperature. The crystal violet solution was then removed, and the samples were washed at least two times with 400 μL sterile dH_2_O to remove any non-adherent dye. Next, 500 μL of 33% glacial acetic acid was added to each well, and then 100 μL of the solution was transferred to a clear 96-well plate with a flat bottom. The optical density was measured at 595 nm using a microplate reader (VICTOR NivoTM HH3500, PerkinElmer^®^, Beaconsfield, UK). The experiment was repeated three times to ensure the validity and reliability of the results. Each trial was conducted using the same methods and procedures as the initial experiment.

#### 2.4.3. Scanning Electron Microscopy (SEM)

To analyze bacterial cell morphology, *Streptococcus oralis* was cultured on BaTiO_3_ piezoelectric ceramic discs for 1 and 2 days (*n* = 1). The samples were washed, fixed with 1.5% glutaraldehyde, and dehydrated using increasing ethanol concentrations (20%, 30%, 40%, 50%, 60%, 70%, 80%, 90% and 100%) with 15 min incubation interval. Afterwards, all the samples were coated with a 15 nm ultrathin gold–palladium film (80–20% in weight). SEM was conducted at different magnifications (10 Kv × 5 Kv, 10 μm) and bacterial morphology, interactions with material and bacterial/biofilm density were evaluated by two calibrated observers.

#### 2.4.4. Confocal Laser Scanning Microscopy (CLSM)

The LIVE/DEAD fluorescent staining *Streptococcus oralis* biofilms grown on BaTiO_3_ piezoelectric and control ceramic discs was assessed after 1 day of culture (*n* = 1). The bacterial cell samples were washed and stained with Propidium Iodide (PI, 535/617)-staining dead cells and DAPI (358/461)-staining all cells for approximately 20 min, while protecting them from light. Discs were then washed again before being observed using CLSM. CLSM images were analyzed using FIJI software, and 3D reconstructions of the stack images obtained were produced. Image analysis covered the LIVE/DEAD cell ratio for each group.

### 2.5. Statistical Analysis

Statistical analysis was performed using IBM^®^ SPSS^®^ 27.0 statistics software for Mac (SPSS, Chicago, IL, USA). Data was tested for normality by Kolmogorov–Smirnov test. Comparisons between groups for roughness values, cell viability, cell average area, ALP activity, interleukins 1β and 6, osteopontin levels, CFU and biomass were carried out using a factorial analysis of variance ANOVA or Kruskal–Wallis tests as appropriate, with Tukey’s post-hoc test to identity groups with significant differences. Significance level was set as *p* < 0.05. All data is presented as mean ± standard deviation (SD).

## 3. Results

### 3.1. Sample Characterization

#### 3.1.1. Microstructure

**Figure 1:**
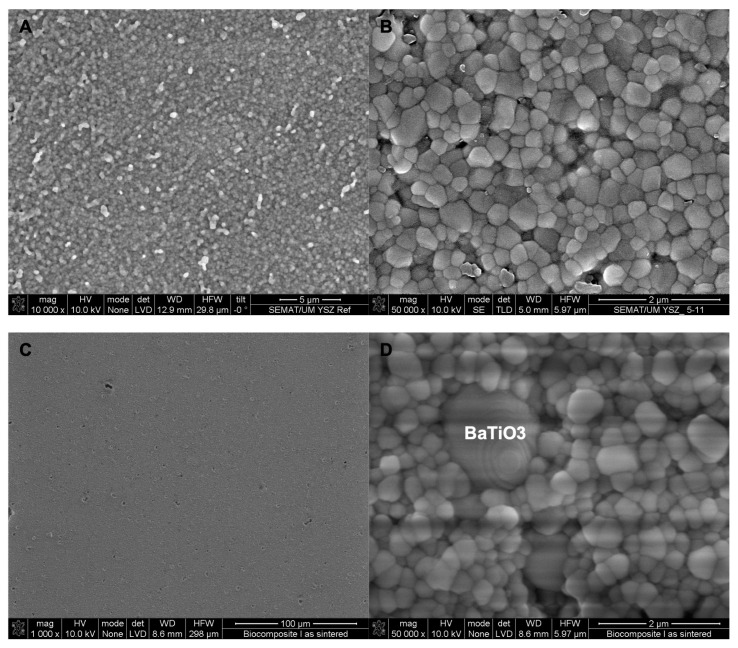
BSE/SEM micrographs of sintered YSZ (**A**,**B**) and YSZ with 5 wt.% BaTiO_3_ (**C**,**D**) samples. 1000× magnification (**C**), 10,000× magnification (**A**) and 50,000× magnification (**B**,**D**).

Before contact poling and cell cultures were undertaken, BSE/SEM images were obtained (Figure 1). BSE/SEM images showed that the BaTiO_3_ composite samples have higher porosity and particle pull-outs at the surface compared with YSZ surfaces. The distribution of BaTiO_3_ grains in 5% BaTiO_3_/YSZ samples were shown to be homogenous. YSZ grain size 0.25 μm was the same in reference samples and in the 5% BaTiO_3_/YSZ samples and the BaTiO_3_ grain average diameter was 2.64 μm ± 1.59 μm. Poling procedures did not affect surface properties and morphology.

#### 3.1.2. Surface Roughness

Before contact poling, surface roughness was evaluated using Ra parameters and results are tabulated in Table 2, presented as mean and standard deviation (SD). The results showed similar Ra values between groups, with no significant differences (*p* > 0.05). However, Ra values of sintered samples before polishing were higher than the Ra values of polished samples.

### 3.2. Cell Culture

#### 3.2.1. Cell Viability

**Figure 2:**
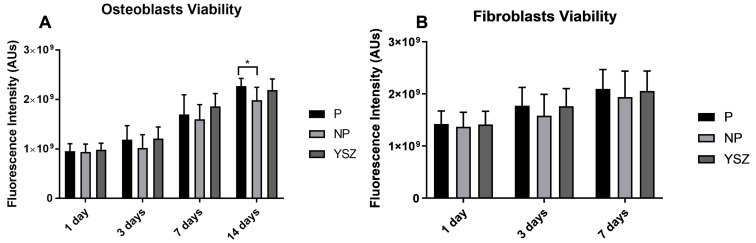
Bar charts showing osteoblasts (**A**) and fibroblasts (**B**) viability measured as mean using fluorescence intensity expressed in arbitrary resorufin units. Error bars represent standard deviation. Statistical significance: * *p* < 0.05, *one-way* ANOVA.

Cell viability results were obtained for 1, 3, 7 and 14 days for osteoblasts and fibroblasts (shown in Figure 2A,B, respectively). Osteoblasts viability increased over time for all groups without significant differences between groups (*p* > 0.05, *one-way ANOVA repeated measures*), however significantly higher values were observed on the poled group compared to the non-poled group at 14 days (*p* < 0.05, *one-way* ANOVA). No differences were found in fibroblasts viability between groups over time (*p* > 0.05, *one-way ANOVA repeated measures)* and in determined time-points (*p* > 0.05, *one-way ANOVA*).

#### 3.2.2. Cell Morphology

**Figure 3:**
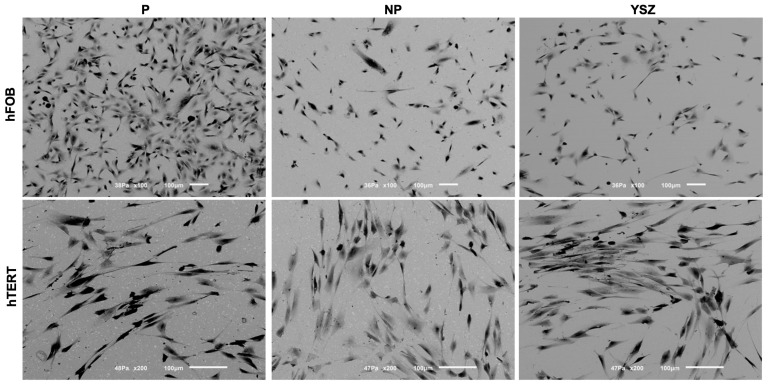
BSE/SEM micrographs with 100× magnification of osteoblasts (hFOB) and 200× magnification of fibroblasts (hTERT) cultured on surfaces at 1 day.

**Figure 4:**
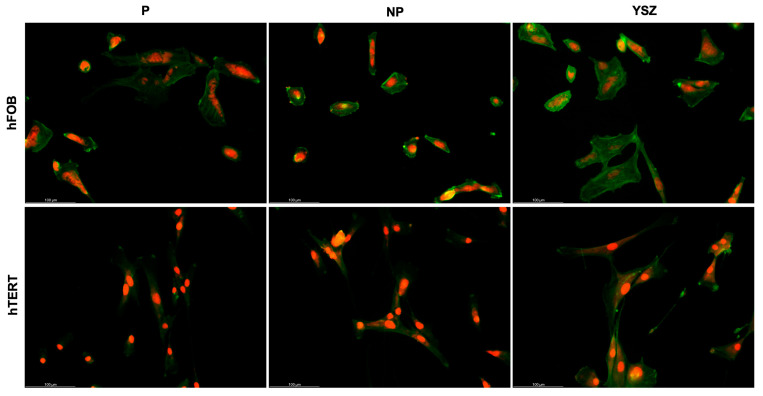
Fluorescence microscopy images of osteoblasts (hFOB) and fibroblasts (hTERT) on samples after 1 day of culture. Cytoskeleton staining (green) and cell nucleic acid staining (red). Scale bar = 100 μm.

BSE/SEM (Figure 3) and fluorescent images (Figure 4) obtained on samples after 24 h of culturing of osteoblasts and fibroblasts are presented with the respective magnifications. BSE/SEM micrographs and fluorescent images showed adherent cells in all groups after 1 day. Poled samples seem to have osteoblasts with irregular morphology, higher cell spreading covering a larger area when compared to non-poled and YSZ samples. Fibroblasts distribution appears more homogeneous and fluorescent images revealed a pattern of nucleus and cytoskeleton alignment that is more evident on poled discs.

#### 3.2.3. ALP Activity

**Figure 5:**
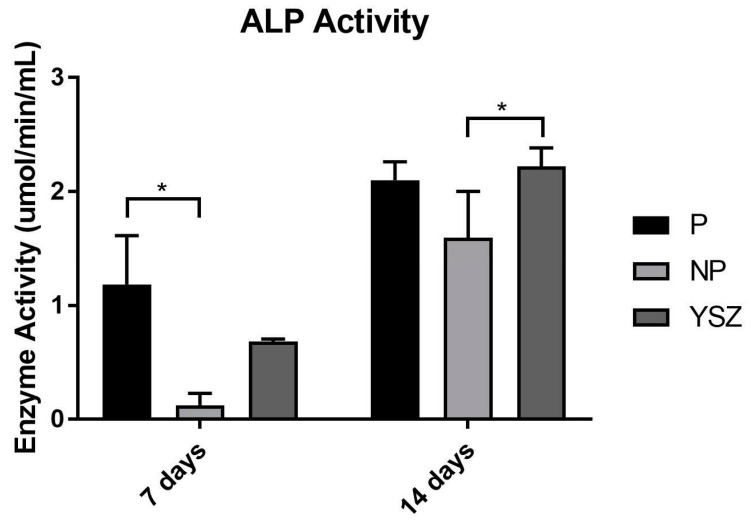
Bar chart showing ALP activity as mean and standard deviation of enzymatic activity in μmol/min/mL. Error bars represent standard deviation. Statistical significance: * *p* < 0.05, *one-way* ANOVA *and one-away ANOVA repeated measures*.

ALP results based on osteoblasts suspension at 7 and 14 days of culture are shown in Figure 5. Despite ALP activity increasing from 7 to 14 days in all groups, NP group presented a significant higher increase comparing to YSZ (*p* < 0.05, *one-way ANOVA repeated measures*). ALP activity was significantly increased in the poled group at 7 days and YSZ group at 14 days compared to the non-poled group, respectively (*p* < 0.05, *one-way* ANOVA).

#### 3.2.4. Interleukin 1β

**Figure 6:**
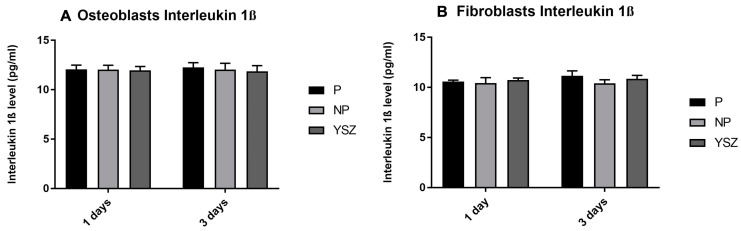
Bar charts showing osteoblasts (**A**) and fibroblasts (**B**) interleukin 1β as mean concentration in pg/mL. Error bars represent standard deviation.

The analysis of interleukin 1β secretion by the osteoblasts and fibroblasts cultures in all groups under study was performed at 1 and 3 days, as shown in Figure 6. The results obtained show constant IL-1β secretion over time and similar values between groups and cell types, without statistically significant differences (*p* > 0.05, *one-way* ANOVA and *one-wat ANOVA repeated measures*).

#### 3.2.5. Interleukin 6

**Figure 7:**
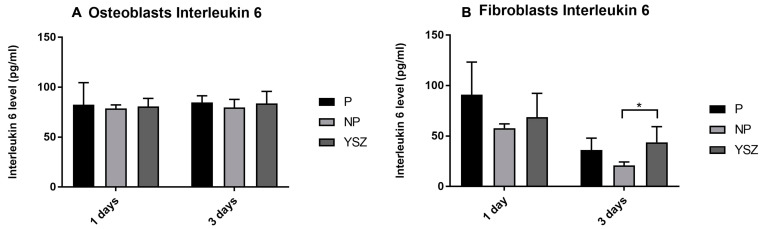
Bar charts showing osteoblasts (**A**) and fibroblasts (**B**) interleukin 6 as mean concentration in pg/mL. Error bars represent standard deviation. Statistical significance: * *p* < 0.05, *one-way* ANOVA.

Interleukin 6 secretion by osteoblasts and fibroblasts was obtained at 1 and 3 days, as presented in Figure 7. The results revealed no statistically significant differences between groups on osteoblasts culture in both measured times (*p* > 0.05, *one-way* ANOVA and *one-way ANOVA repeated measures*). Fibroblasts IL-6 secretion decreased from 1 to 3 days in all study groups with significant lower values in non-poled group comparing to YSZ at 3 days (*p* < 0.05, *one-way* ANOVA). However, repeated measures analysis revealed no significant differences between groups over time (*p* > 0.05, *one-way ANOVA repeated measures*).

#### 3.2.6. Osteopontin

**Figure 8:**
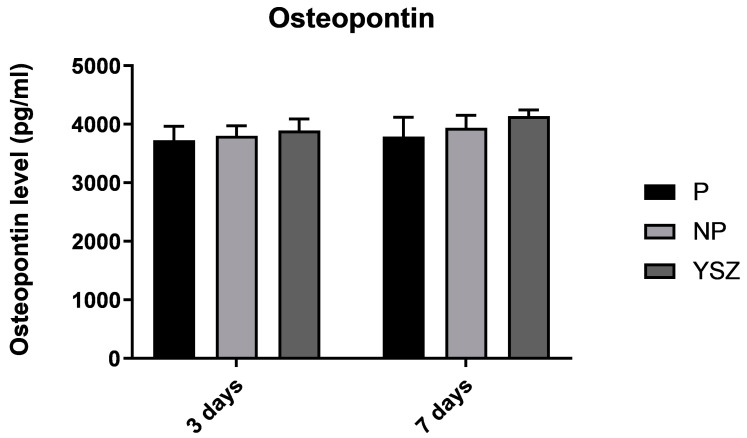
Bar charts showing osteopontin levels on osteoblasts culture as mean concentration in pg/mL. Error bars represent standard deviation.

Osteopontin concentration slightly increased over time in all groups (Figure 8). Although higher ALP activity in the poled group at 7 days and YSZ group at 14 days compared to the non-poled group was observed, no significant differences in osteopontin secretion were observed between groups (*p* > 0.05, *one-way* ANOVA and *one-way ANOVA repeated measures*).

#### 3.2.7. Cell Mineralization

**Figure 9:**
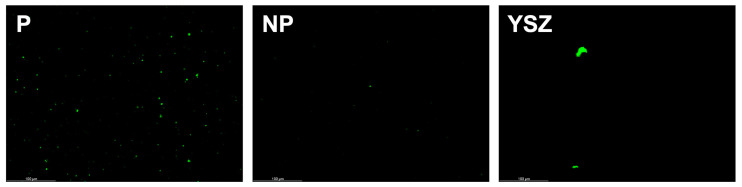
Fluorescence microscopy images obtained by osteoimage-stained method at 7 days of osteoblasts culture. Bone mineral nodules on poled (P), non-poled (NP) and zirconia [31] samples. Scale bar = 100 μm.

Specific staining of hydroxyapatite mineralization nodules by OsteoImage at 7 days in osteoblast culture is shown in Figure 9. A higher density in hydroxyapatite nodule formation was detected on poled surfaces for 7 days of cell culture compared to non-poled and reference YSZ samples.

### 3.3. Bacterial Growth

#### 3.3.1. Colony Forming Unit

**Figure 10:**
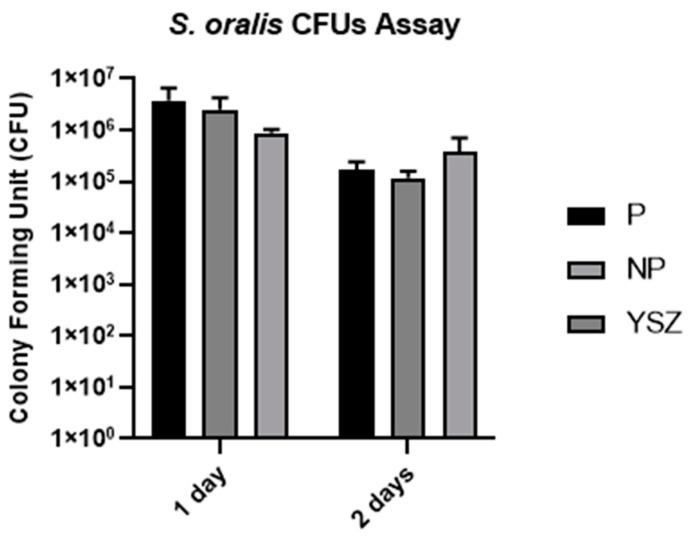
Bar charts showing *Streptococcus oralis* CFUs measured as mean ± standard deviation. Error bars represent standard deviation.

CFUs of *Streptococcus oralis* were evaluated at two different time points—1 and 2 days of culture—and it was observed that the CFU per milliliter decreased from the first day to the second day in all study groups, as shown in Figure 10. Multiple comparisons between the groups at the two timepoints assessed revealed no statistically significant differences (*p* > 0.05, *one-way* ANOVA and *one-way ANOVA repeated measures*).

#### 3.3.2. Biomass

**Figure 11:**
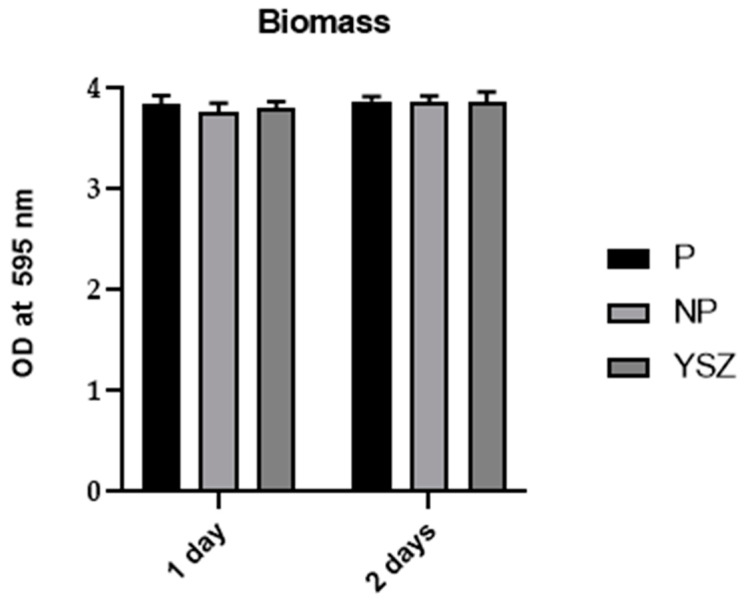
Bar charts showing *Streptococcus oralis* biomass measured as mean ± standard deviation using violet crystal 1%. Error bars represent standard deviation.

The results of biomass of *Streptococcus oralis* were observed to be similar on the first and second day of culture, as shown in Figure 11. Additionally, a repeated-measures ANOVA was conducted and no significant differences between study groups were observed on both time points (*p* > 0.05, *one-way* ANOVA and *one-way ANOVA repeated measures*).

#### 3.3.3. Scanning Electron Microscopy (SEM)

**Figure 12:**
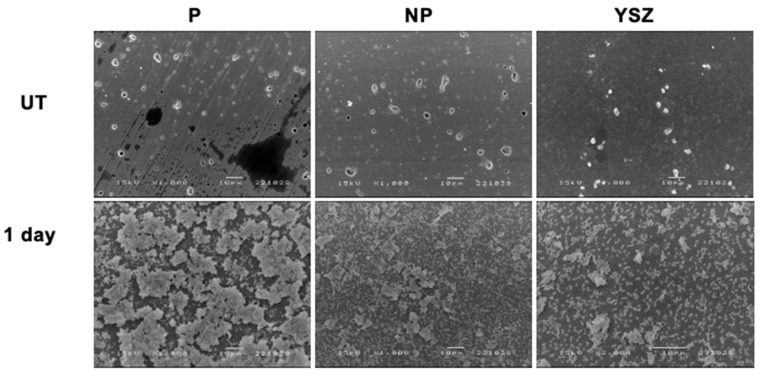
Scanning electron microscopy images obtained at 1 day of *S. oralis* culturing with 1000× magnification. Scale bar = 10 μm. UT—baseline samples; 1 day—samples after 1 day of *S. oralis* culturing.

The Scanning Electron Microscopy images in the Figure 12 describe *Streptococcus oralis* cultured on samples. When observed after one day of culture, the images revealed standard spherical bacterial cells, characteristic of cocci, present in all samples. Interestingly, the P group showed a perceived higher amount of bacteria compared to the NP and YSZ groups.

#### 3.3.4. Confocal Laser Scanning Microscopy (CLSM)

**Figure 13:**
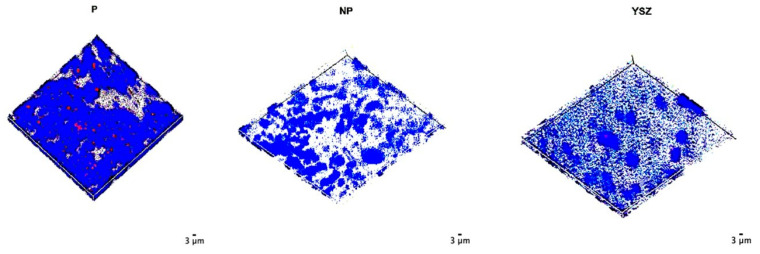
Confocal laser scanning microscopy 3D images obtained at 1 day of *Streptococcus oralis* culture. Scale bar = 80 mm.

The CLSM images presented in Figure 13 were captured after one day of culturing *Streptococcus oralis* culture on the samples. It was apparent that the bacterial growth was present in all samples, with the P group displaying the greatest amount of growth compared to the NP and YSZ groups. Upon examining the images, it was found that the DAPI maker had stained most bacterial cells, rather than IP maker, thus demonstrating the presence of viable, live bacteria.

## 4. Discussion

Implant surfaces properties can affect the biological behavior of peri-implant tissues. Based on this, surface treatments have been used to stimulate osteoblasts activity, extracellular matrix deposition and bone mineralization [1,3,10,11]. This has led to the continued search for optimal implant surfaces to minimize the osseointegration time and healing period [3,4,6,11]. A possible approach is based on the use of electrically stimulated surfaces, such as BaTiO_3_, one of the most thoroughly studied material with piezoelectric properties [32] in order to accelerate the osseointegration process [3,4,11,19,20,21].

In this context, this in vitro study suggests the use of BaTiO_3_ piezoelectric ceramic as implant material and aims to understand the effect of these surfaces on the biological response of human gingival fibroblasts, human fetal osteoblasts and *Streptococcus oralis* adhesion.

Cell viability increased over time for all groups in both cell types (fibroblasts and osteoblasts) suggesting that zirconia composite surfaces with the addition of BaTiO_3_ are not cytotoxic to these cells. Poled zirconia surfaces with 5% BaTiO_3_ demonstrated superior results of osteoblast viability with significantly higher values compared to non-poled surfaces at 14 days of culture (*p* < 0.05). The obtained results are in line with previous studies carried out with bone marrow cells [5,21], mesenchymal stem cells [22], human umbilical vein endothelial cells and human primary osteoblasts [11], and osteoblast-like cells derived from osteosarcoma [33]. Additionally, the results of an in vitro study with mouse osteoblast cell line showed that BaTiO_3_ foam did not present higher significantly cytotoxicity when compared to bioglass [29]. In contrast, fibroblasts viability does not seem to be influenced by polarization, which is in accordance with other study performed with primary fibroblasts [34]. We propose that the significant higher osteoblast viability in poled group can be explained by the presence of a positive charge on the sample surface attracting ions that will contribute to increasing growth factors and subsequently bone mineralization [35].

The morphological analysis with SEM/BSE and fluorescent microscopy demonstrated that cells cultured on discs after 24 h were adherent. Poled samples showed osteoblasts with prismatic conformation, evidencing the filopodia formation, in greater quantity and more spread when compared to non-poled and YSZ groups. Despite the fibroblast distribution seemed more homogeneous between groups, cells cultured on poled group discs adopted a more evident pattern of alignment of nucleus and cytoskeleton. These observations, consistent with other results in the literature [3,22,34], could contribute to the extracellular matrix production and cell differentiation, since cytoskeleton alignment, as a function of cell-material contact, induces transcription regulation of differentiation-related genes [36].

Alkaline phosphatase was used in this study as a differentiation marker for osteoblasts [37,38]. ALP activity increased in all groups from 7 to 14 days, however it was significantly higher in the poled group at 7 days compared to the non-poled group, which is consistent with previous studies [21,33]. Additionally, a significantly higher activity was found in YSZ group compared to the non-poled group at 14 days of culture. This result implies that the non-poled group had the lower functional osteoblastic activity. Considering the results of ALP activity, and when observing the hydroxyapatite mineralization nodules, represented by the fluorescence signal, the density is apparently higher on poled samples. The production of osteopontin, a sialoprotein component of the bone matrix synthesized by osteoblasts, was also evaluated in this study as a differentiation marker for osteoblasts [39]. Osteopontin concentration slightly increased from 3 to 7 days of culture, but no significant differences were found between groups. Higher values would have been expected for the poled group as they present higher osteoblasts viability, ALP activity and hydroxyapatite nodules formation; however, osteopontin is a marker of late differentiation and the measure was performed at 3 and 7 days. In this sense, the measurement of this protein at a later stage may be considered in future analyses.

Regarding inflammatory markers, lower values of interleukin 1β would be expected for osteoblasts in contact with poled samples, as it had higher cellular viability and differentiation, represented by the ALP activity and hydroxyapatite nodule formation results. However, interleukin 1β concentration was very similar in all groups without significant differences between them for both cell cultures. These results may be explained by the fact that the measurements were carried out only in an initial phase of cell culture. Probably, if measurement at later time-points was considered, differences between groups could have been detected [39]. Osteoblast secretion of interleukin 6 remained constant, however fibroblast secretion decreased in all groups from 1 to 3 days of culture and significantly lower values were observed in the non-poled group compared to YZS at 3 days in the poled group. This fact suggests the possible anti-inflammatory role of non-poled BaTiO_3_ mediated by IL-6 on fibroblasts activity. This result is in accordance with an in vitro study by Majumdar et al. [40] with human erythromyeloma cells that suggest that barium-doped bioactive glass induces an amelioration of the inflammatory response by cytokine homeostasis, alleviating the production of pro-inflammatory cytokines and simultaneously stimulating the secretion of anti-inflammatory mediators. These results could be justified by the fact that fibroblasts in contact with non-poled samples show lower viability values as well as IL-1β secretion compared to the other groups. Cytoskeleton alignment in poled samples, as discussed previously, could be a potential regulation mechanism inducing lower IL-6 transcription and secretion and resulting in a potential lower inflammation mediated by fibroblasts in poled samples.

The effectiveness of BaTiO_3_ as an antibiofilm agent against *Streptococcus oralis* culture was evaluated by measuring biofilm biomass and metabolic activity (by counting the number of viable cells) after 1 and 2 days of culture. The results showed that there were no statistically significant differences in CFUs and biomass between the study and comparison groups over time. However, a decrease in CFUs from 1 to 2 days of culture was observed, suggesting an impact of BaTiO_3_ on the biofilm formation. Although the literature describes that zirconia implant surfaces can lead to a significant decrease in the adhesion of periodontal microorganisms in vitro when compared to titanium implant surfaces [41], this results also suggest that prolonged exposure to BaTiO_3_ may reduce CFUs in *Streptococcus oralis* without causing a bactericidal effect. The rationale for using *S. oralis* was its role as a primary colonizer as widely described in the literature [42,43]. While the specific inflammatory events that culminate in the onset of peri-implant disease are driven by other bacterial strains, the colonization process of implant surfaces is always initiated by primary colonizers and pathological species depend on this initial colonization to be able to co-aggregate and turn the biofilm into a more mature form. The use of *S. oralis* as a model of early colonization of implant surfaces is well-established [42,43]; however, the available evidence on BaTiO_3_ is limited to other bacterial species, namely *Pseudomonas aeruginosa*, *Staphylococcus aureus* [24] and *Streptococcus mutans* [28] which are in line with the results of our study.

Moreover, the piezoelectric agent that was added to the samples—BaTiO_3_—seemed to favor the initial formation of biofilms, but this effect did not persist throughout the entire study period, as observed in the CFU assay. The results suggest that the piezoelectric properties accelerate the initial biofilm formation but not the biofilm maturation process, however these results need to be further confirmed. This behavior was also observed in confocal laser scanning microscopy and scanning electron microscopy images after 1 day of *S. oralis* culture.

The results of this study suggest that the addition of BaTiO_3_ is not cytotoxic to peri-implant cells and highlight the potential effect of BaTiO_3_ zirconia implant surfaces with or without piezoelectric properties on osteoblasts and fibroblasts cells, respectively.

In our study, we introduced a zirconia surface with dispersed particles of barium titanate with acceptable mechanical properties [30]. However, studies about the processing method and possible texturing should be further investigated and improved mechanical and surface characterization methods should be included, such as optical 3D profilometry.

Finally, this was an in vitro study, and the inferences from in vitro generated data are limited, therefore in vivo studies should be performed based on the results of this study. Also, further studies should be carried out to assess the piezoelectric effect of these materials on peri-implant tissues when load is applied.

## 5. Conclusions

The results of this study suggest that zirconia composite surfaces with the addition of piezoelectric BaTiO_3_ are not cytotoxic to peri-implant tissues cells and seem to promote a faster initial osteoblast differentiation. Additionally, samples with or without piezoelectric properties did not affect osteoblast inflammatory profile. Nevertheless, the addition of non-poled BaTiO_3_ to zirconia may have a potential reduction effect in IL-6 mediated inflammatory activity of fibroblasts. In addition, the study observed that prolonged exposure to BaTiO_3_ ceramics can induce a bacteriostatic behavior in *Streptococcus oralis*. However, the piezoelectric properties of BaTiO_3_ did not seem to have a bacteriostatic or bactericidal effect on *Streptococcus oralis.*

## Figures and Tables

**Table 1:** Sample groups and composition.

Designation	Composition
Poled (P)	YSZ with 5 wt.% BaTiO_3_ polarized
Non-poled (NP)	YSZ with 5 wt.% BaTiO_3_ non-polarized
YSZ	3% Yttria-stabilized zirconia

**Table 2:** Samples contact roughness (Ra) measured with Archimedes method.

Sample	Ra (μm)	SD	Notes
5% BaTiO_3_/YSZ	0.47 *	0.10	as sintered
	0.05 **	0.03	after polishing
YSZ	0.50 *	0.10	as sintered
	0.04 **	0.08	after polishing

*as sintered, ** after polishing.

## Data Availability

Not applicable.

## References

[1] Ferreira Ribeiro C., Cogo-Muller K., Franco G.C., Silva-Concilio L.R., Sampaio Campos M., de Mello Rode S., Claro Neves A.C. (2016). Initial oral biofilm formation on titanium implants with different surface treatments: An in vivo study. Arch. Oral. Biol..

[2] Cionca N., Hashim D., Mombelli A. (2017). Zirconia dental implants: Where are we now, and where are we heading?. Periodontology 2000.

[3] Pettersen E., Shah F.A., Ortiz-Catalan M. (2021). Enhancing osteoblast survival through pulsed electrical stimulation and implications for osseointegration. Sci. Rep..

[4] Pettersen E., Anderson J., Ortiz-Catalan M. (2022). Electrical stimulation to promote osseointegration of bone anchoring implants: A topical review. J. Neuroeng. Rehabil..

[5] Furuya K., Morita Y., Tanaka K., Katayama T., Nakamachi E. Acceleration of osteogenesis by using barium titanate piezoelectric ceramic as an implant material. Proceedings of the Bioinspiration, Biomimetics, and Bioreplication.

[6] von Wilmowsky C., Moest T., Nkenke E., Stelzle F., Schlegel K.A. (2014). Implants in bone: Part I. A current overview about tissue response, surface modifications and future perspectives. Oral. Maxillofac. Surg..

[7] Cho Y.D., Shin J.C., Kim H.L., Gerelmaa M., Yoon H.I., Ryoo H.M., Kim D.J., Han J.S. (2014). Comparison of the osteogenic potential of titanium- and modified zirconia-based bioceramics. Int. J. Mol. Sci..

[8] Wenz H.J., Bartsch J., Wolfart S., Kern M. (2008). Osseointegration and clinical success of zirconia dental implants: A systematic review. Int. J. Prosthodont..

[9] Kuroda K., Okido M. (2012). Hydroxyapatite coating of titanium implants using hydroprocessing and evaluation of their osteoconductivity. Bioinorg. Chem. Appl..

[10] Hirano T., Sasaki H., Honma S., Furuya Y., Miura T., Yajima Y., Yoshinari M. (2015). Proliferation and osteogenic differentiation of human mesenchymal stem cells on zirconia and titanium with different surface topography. Dent. Mater. J..

[11] Poon K.K., Wurm M.C., Evans D.M., Einarsrud M.A., Lutz R., Glaum J. (2020). Biocompatibility of (Ba,Ca)(Zr,Ti)O_3_ piezoelectric ceramics for bone replacement materials. J. Biomed. Mater. Res. B Appl. Biomater..

[12] Ahn A.C., Grodzinsky A.J. (2009). Relevance of collagen piezoelectricity to “Wolff’s Law”: A critical review. Med. Eng. Phys..

[13] Nakamura M., Hiratai R., Yamashita K. (2012). Bone mineral as an electrical energy reservoir. J. Biomed. Mater. Res. A.

[14] Marino A., Becker R.O. (1970). Piezoelectric effect and growth control in bone. Nature.

[15] Dergin G., Akta M., Gursoy B., Devecioglu Y., Kurkcu M., Benlidayi E. (2013). Direct current electric stimulation in implant osseointegration: An experimental animal study with sheep. J. Oral. Implantol..

[16] Fukada E., Yasuda I. (1957). On the Piezoelectric Effect of Bone. J. Phys. Soc. Jpn..

[17] Bassett C.A., Becker R.O. (1962). Generation of electric potentials by bone in response to mechanical stress. Science.

[18] Bassett C.A.L., Pawluk R.J., Becker R.O. (1964). Effects of Electric Currents on Bone In Vivo. Nature.

[19] Song J.K., Cho T.H., Pan H., Song Y.M., Kim I.S., Lee T.H., Hwang S.J., Kim S.J. (2009). An electronic device for accelerating bone formation in tissues surrounding a dental implant. Bioelectromagnetics.

[20] Salman N.N., Park J.B. (1980). The effect of direct electrical current stimulation on the bone/porous metallic implant interface. Biomaterials.

[21] Fan B., Guo Z., Li X., Li S., Gao P., Xiao X., Wu J., Shen C., Jiao Y., Hou W. (2020). Electroactive barium titanate coated titanium scaffold improves osteogenesis and osseointegration with low-intensity pulsed ultrasound for large segmental bone defects. Bioact. Mater..

[22] Rocca A., Marino A., Rocca V., Moscato S., de Vito G., Piazza V., Mazzolai B., Mattoli V., Ngo-Anh T.J., Ciofani G. (2015). Barium titanate nanoparticles and hypergravity stimulation improve differentiation of mesenchymal stem cells into osteoblasts. Int. J. Nanomed..

[23] Swain S., Padhy R.N., Rautray T.R. (2020). Polarized piezoelectric bioceramic composites exhibit antibacterial activity. Mater. Chem. Phys..

[24] Shah A.A., Khan A., Dwivedi S., Musarrat J., Azam A. (2018). Antibacterial and Antibiofilm Activity of Barium Titanate Nanoparticles. Mater. Lett..

[25] Swain S., Rautray T.R. (2021). Assessment of Polarized Piezoelectric SrBi_4_Ti_4_O_15_ Nanoparticles as an alternative antibacterial agent. bioRxiv.

[26] Marin E., Boschetto F., Sunthar T.P.M., Zanocco M., Ohgitani E., Zhu W., Pezzotti G. (2021). Antibacterial effects of barium titanate reinforced polyvinyl-siloxane scaffolds. Int. J. Polym. Mater. Polym. Biomater..

[27] Pang S., He Y., Zhong R., Guo Z., He P., Zhou C., Xue B., Wen X., Li H. (2019). Multifunctional ZnO/TiO_2_ nanoarray composite coating with antibacterial activity, cytocompatibility and piezoelectricity. Ceram. Int..

[28] Montoya C., Jain A., Londoño J.J., Correa S., Lelkes P.I., Melo M.A., Orrego S. (2021). Multifunctional Dental Composite with Piezoelectric Nanofillers for Combined Antibacterial and Mineralization Effects. ACS Appl. Mater. Interfaces.

[29] Ball J.P., Mound B.A., Nino J.C., Allen J.B. (2014). Biocompatible evaluation of barium titanate foamed ceramic structures for orthopedic applications. J. Biomed. Mater. Res. A.

[30] Tiainen L.K. (2021). Design, Manufacturing & Analysis of Smart Ceramics for Biomedical Applications: Zirconia Functionalization with Barium Titanate. Ph.D. Thesis.

[31] Khare D., Basu B., Dubey A.K. (2020). Electrical stimulation and piezoelectric biomaterials for bone tissue engineering applications. Biomaterials.

[32] Swain S., Bowen C., Rautray T. (2021). Dual response of osteoblast activity and antibacterial properties of polarized strontium substituted hydroxyapatite-Barium strontium titanate composites with controlled strontium substitution. J. Biomed. Mater. Res. A.

[33] Wang A., Liu Z., Hu M., Wang C., Zhang X., Shi B., Fan Y., Cui Y., Li Z., Ren K. (2018). Piezoelectric nanofibrous scaffolds as in vivo energy harvesters for modifying fibroblast alignment and proliferation in wound healing. Nano Energy.

[34] Lirani A.P.R., Lazaretti-Castro M. (2005). Evidências da ação de agentes físicos sobre o metabolismo do tecido ósseo e seus potenciais usos clínicos. Arq. Bras. Endocrinol. Endocrinol. Metabol..

[35] Mathieu PS L.E. (2012). Cytoskeletal and Focal Adhesion Influences on Mesenchymal Stem Cell Shape, Mechanical Properties, and Differentiation Down Osteogenic, Adipogenic, and Chondrogenic Pathways. Tissue Eng. Part B Rev..

[36] Fernandes B.F., da Cruz M.B., Marques J.F., Madeira S., Carvalho O., Silva F.S., da Mata A., Carames J.M.M. (2020). Laser Nd:YAG patterning enhance human osteoblast behavior on zirconia implants. Lasers Med. Sci..

[37] Peñarrieta-Juanito G., Cruz M., Costa M., Miranda G., Marques J., Magini R., Mata A., Souza J.C.M., Caramês J., Silva F.S. (2018). A novel gradated zirconia implant material embedding bioactive ceramics: Osteoblast behavior and physicochemical assessment. Materialia.

[38] da Cruz M.B., Marques J.F., Fernandes B.F., Pinto P., Madeira S., Carvalho O., Silva F.S., Carames J.M.M., da Mata A. (2022). Laser surface treatment on Yttria-stabilized zirconia dental implants: Influence on cell behavior. J. Biomed. Mater. Res. B Appl. Biomater..

[39] Majumdar S., Hira S.K., Tripathi H., Kumar A.S., Manna P.P., Singh S.P., Krishnamurthy S. (2021). Synthesis and characterization of barium-doped bioactive glass with potential anti-inflammatory activity. Ceram. Int..

[40] Lorusso F., Noumbissi S., Francesco I., Rapone B., Khater A.G.A., Scarano A. (2020). Scientific Trends in Clinical Research on Zirconia Dental Implants: A Bibliometric Review. Materials.

[41] Ingendoh-Tsakmakidis A., Eberhard J., Falk C.S., Stiesch M., Winkel A. (2020). In Vitro Effects of Streptococcus oralis Biofilm on Peri-Implant Soft Tissue Cells. Cells.

[42] D’Ercole S., Di Campli E., Pilato S., Iezzi G., Cellini L., Piattelli A., Petrini M. (2021). Streptococcus oralis Biofilm Formation on Titanium Surfaces. Int. J. Oral. Maxillofac. Implant..

[43] D’Ercole S., Cellini L., Pilato S., Di Lodovico S., Iezzi G., Piattelli A., Petrini M. (2020). Material characterization and Streptococcus oralis adhesion on Polyetheretherketone (PEEK) and titanium surfaces used in implantology. J. Mater. Sci. Mater. Med..

